# Sarcoma risk after radiation exposure

**DOI:** 10.1186/2045-3329-2-18

**Published:** 2012-10-04

**Authors:** Amy Berrington de Gonzalez, Alina Kutsenko, Preetha Rajaraman

**Affiliations:** 1 Radiation Epidemiology Branch, Division of Cancer Epidemiology & Genetics, NCI/NIH, 6120 Executive Boulevard (Rm 7034), Bethesda, MD 20892, USA

## Abstract

Sarcomas were one of the first solid cancers to be linked to ionizing radiation exposure. We reviewed the current evidence on this relationship, focusing particularly on the studies that had individual estimates of radiation doses. There is clear evidence of an increased risk of both bone and soft tissue sarcomas after high-dose fractionated radiation exposure (10 + Gy) in childhood, and the risk increases approximately linearly in dose, at least up to 40 Gy. There are few studies available of sarcoma after radiotherapy in adulthood for cancer, but data from cancer registries and studies of treatment for benign conditions confirm that the risk of sarcoma is also increased in this age-group after fractionated high-dose exposure. New findings from the long-term follow-up of the Japanese atomic bomb survivors suggest, for the first time, that sarcomas can be induced by acute lower-doses of radiation (<5 Gy) at any age, and the magnitude of the risk is similar to that observed for other solid cancers. While there is evidence that individuals with certain rare familial genetic syndromes predisposing to sarcoma, particularly Nijmegen Breakage Syndrome, are particularly sensitive to the effects of high dose radiation, it is unclear whether this is also true in very low-dose settings (<0.1 Gy). The effects of common low-penetrance alleles on radiosensitivity in the general population have not been well-characterized. Some evidence suggests that it may be possible to identify radiation-induced sarcomas by a distinct molecular signature, but this work needs to be replicated in several dose settings, and the potential role of chemotherapy and tumor heterogeneity needs to be examined in more detail. In summary, radiation exposure remains one of the few established risk factors for both bone and soft tissue sarcomas. Similar to many other cancers children have the highest risks of developing a radiation-related sarcoma. Efforts to limit unnecessary high-dose radiation exposure, particularly in children, therefore remain important given the high fatality rates associated with this disease.

##  

Sarcomas are a rare but highly fatal outcome of radiation exposure. The first case reports of bone sarcomas in patients who had received radiotherapy for benign bone conditions were published as early as 1922 [1], making it one of the first solid cancers to be linked to radiation. Martland’s famous report of bone sarcomas in the jaws of radium-dial painters followed in 1929 [2]. In the last few decades much has been learned about the relationship by studying the occurrence of sarcomas after radiotherapy treatment for both benign and malignant diseases. Nevertheless, because radiation-related sarcoma is a very rare event, studies have generally been small, and many questions remain about the relationship. Uncertainties include the shape of the dose–response relationship, particularly at lower (<5 Gy (Gray)) and very high absorbed doses (20 + Gy), the impact of factors such as age at exposure, time since exposure, sex and genetic susceptibility on risk as well as understanding variation in risk by sarcoma subtype. Attempts to find a radiation-signature are also currently underway and sarcomas are one of the cancers that have been targeted for these studies.

In this article we review the epidemiological evidence on the association between radiation exposure and development of sarcoma, including both bone and connective tissue sarcomas but differentiating where possible. We focus on studies of radiotherapy for malignant conditions, especially those with individual estimates of absorbed radiation dose to the site of the sarcoma (unless otherwise specified this is the dose that we refer to throughout the article). Treatment of benign conditions has mostly been discontinued and little new data are available since previous reviews [3]. However, we briefly review the findings from these studies and other non-medical populations, especially where there are gaps in the evidence from the studies of treatment for malignant disease. We include assessment of potential effect modifiers including genetic susceptibility and evaluate the studies that have assessed potential radiation-signatures for this disease.

## **High dose radiation exposure (5 + Gy)**

We searched PUBMED using search terms including sarcoma, radiotherapy, dose and subsequent malignancy, and also reviewed the references of included articles. We identified nine studies of radiotherapy treatment and risk of subsequent sarcoma that had estimated the absorbed radiation dose to the site of the sarcoma for each patient [4-11] (Table 1). Six of the studies were of childhood cancers (five included a variety of first cancers and one focused on retinoblastoma); the three studies of adulthood cancer included two studies of cervical cancer and one study of breast cancer survivors. In total these studies included 332 cases of sarcoma and the average dose in the controls varied from 5–27 Gray (Gy). We describe the results below according to age at exposure (childhood versus adulthood) and outcome (bone, soft tissue or both).

**Table 1:** Characteristics of the nine case–control studies of subsequent sarcoma after radiotherapy treatment for cancer with individual dose estimates

					**Average age at diagnosis**		**Dose (controls)**		**ERR/Gy**
**Reference**	**2nd cancer**	**1st cancer**	**Cases**	**Controls**	**1st cancer**	**Sarcoma**	**Average**	**Max**	**(& 95% Cl)**
**Radiotherapy in Childhood**									
Tucker 1987 [4]	Bone Sarcoma	Childhood	68	209	7 yrs	na	27 Gy	60+ Gy	0.06 (0.01–0.20)*
Hawkins 1996 [5]	Bone Sarcoma	Childhood	50	168	ns	na	5 Gy	50+ Gy	0.16 (0.07–0.37)*
Le Vu 1998 [6]	Osteosarcoma	Childhood	32	160	6 yrs	15 yrs	8 Gy	83 Gy	1.4 (0.1–21.8)
Wong 1997 [7]	Soft tissue sarcoma	Retinoblastoma	31	89	<2 yrs	15 yrs	11 Gy	112 Gy	0.17 (0.025–16.3)
Menu-Branthomme 2004 [8]	Soft tissue sarcoma	Childhood	23	111	8 yrs	21 yrs	12 Gy	50 Gy	na
Jenkinson 2007 [9]	Soft tissue sarcoma	Childhood	53	179	7 yrs	17 yrs	5 Gy	30+ Gy	na
**Radiotherapy in Adulthood**									
Boice 1988 [10]	Bone Sarcoma	Cervix	15	155	50 yrs	67 yrs	22 Gy	10+ Gy	0.02 (−0.03–0.21)*
Boice 1988 [10]	Soft tissue sarcoma	Cervix	46	598	50 yrs	67 yrs	7 Gy	10+ Gy	−0.05 (−0.11–0.13)*
Rubino 2005 [11]	All sarcomas	Breast	14	98	55 yrs	62 yrs	19 Gy	80 Gy	0.05 (<0-1.18)

na – not available. ERR/Gy – excess relative risk per Gray. * UNSCEAR [12].

## **Childhood radiotherapy**

**Figure 1:**
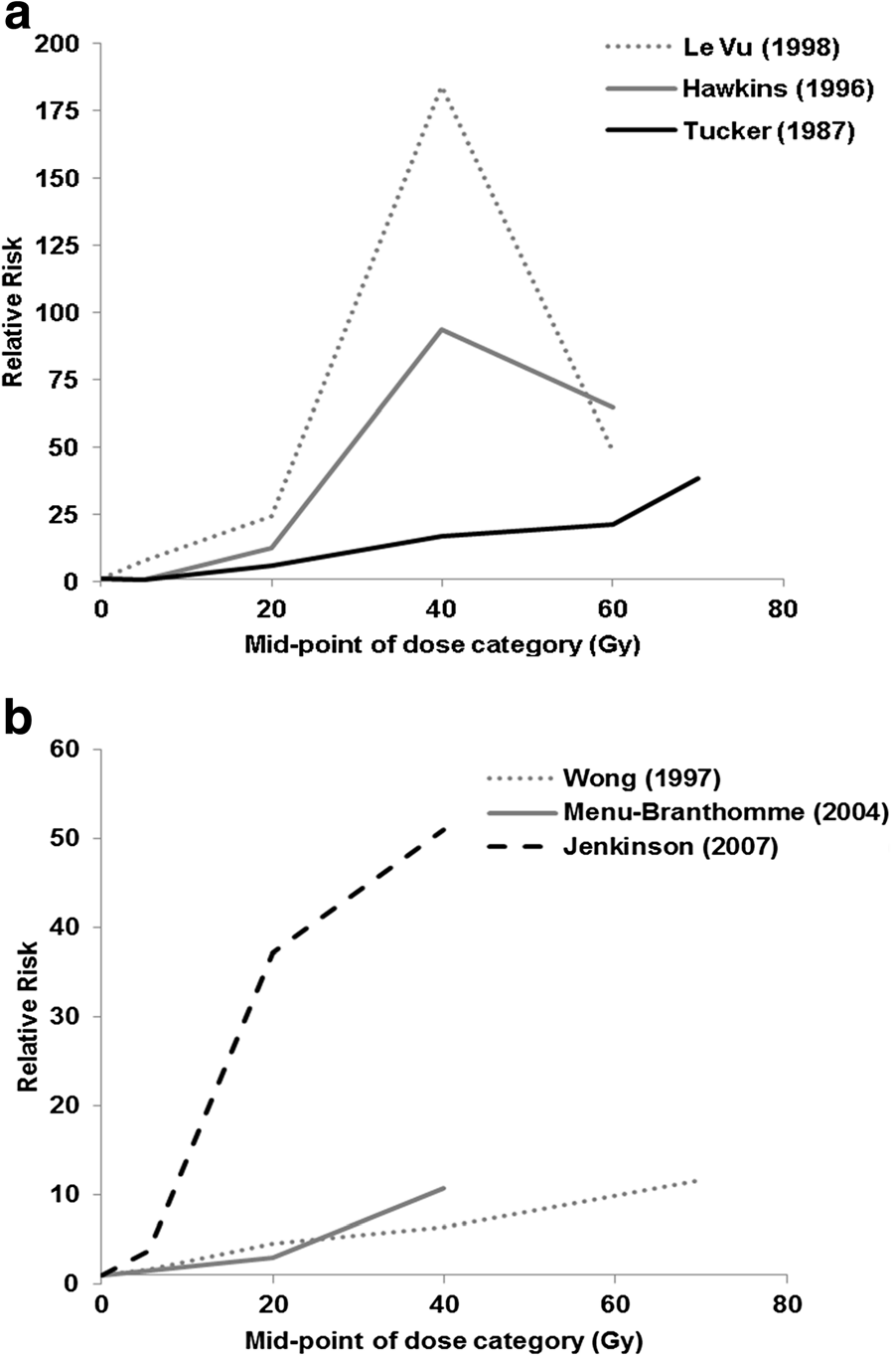
**Relative risk of sarcoma according to radiation dose (Gy) after radiotherapy for childhood cancer. a**) Bone sarcoma. **b**) Soft tissue sarcoma.

There were three studies of *bone* sarcoma after childhood cancer [4-6]. All three studies found that the risk of bone sarcoma increased with increasing radiation dose (Figure 1a). In two of the studies there was evidence that the risk declined after very high doses (40 + Gy) [5,6], which could be the result of high levels of cell killing. For around 20 Gy the relative risk varied from 6 [4] to 24 [6] compared to zero or very low doses of radiation, and in two of the three studies there was little evidence of an excess risk for exposures below 10 Gy, but this could be a result of low statistical power rather than real evidence in support of a threshold (see below for additional comment on lower-dose studies). All three studies included patients with retinoblastoma and these patients are discussed in more detail below as there is evidence of an underlying increased risk of sarcoma in these patients, possibly due to genetic factors.

Of the three studies that examined *soft tissue* sarcoma in relation to radiation dose from radiotherapy treatment [7-9] one consisted only of patients with retinoblastoma [7]. All three studies found an approximately linear relationship between dose and risk, although the magnitude of the relative risk was somewhat lower though than that reported for bone sarcomas (Figure 1b). For a dose of about 20 Gy the relative risk for soft tissue sarcoma was variable: 3 (0.6-14.6) in Menu-Branthomme et al [8], 4.6 (1.7-24.8) in Wong et al. [7], and 37.1 (4.5-309.3) in Jenkinson et al. [9], but the confidence intervals were wide and overlapping. Again there was little evidence of increased risks for doses below 10 Gy, possibly due to low statistical power.

Five of the six studies of childhood radiotherapy assessed the potential interaction between radiotherapy and chemotherapy [4-6,8,9], and four [4-6,8] of these five studies found that the joint effect of radiotherapy and chemotherapy were approximately multiplicative with respect to subsequent risk of sarcoma. Whilst this is consistent with no interaction, from the statistical perspective, it still means that those patients who received both treatmenta often had a very high risk of developing a subsequent sarcoma. In the remaining study [9] the joint effect of the two treatments was additive rather than multiplicative. Most of these studies did not have large enough sample sizes to investigate the impact of different types of chemotherapy in detail, but the most common treatments involved alkylating agents.

Only two studies [6,8] investigated the impact of the radiation dose per fraction. Le Vu et al. [6] found that smaller fractions did decrease the subsequent risk of sarcoma, but Menu-Branthomme et al. [8] did not find any evidence that fraction size affected the risk. The patients in these studies were treated over several decades with a variety of types of radiotherapy including Cobalt-60, orthovoltage and brachytherapy. The type of radiotherapy was taken into account in the estimation of the absorbed dose to the sarcoma site and was not, therefore, routinely investigated separately as a risk factor itself.

## **Adulthood radiotherapy**

Although there was no evidence of an association between radiation dose and *bone* sarcoma in the one study of adulthood exposure with individual dose estimates [10], the study was relatively small (n = 15). Other studies of high-dose radiotherapy, despite having less detailed exposure information do support an association. These include bone marrow transplant patients exposed to high-dose fractions of radiation given as a conditioning regimen [13], ankylosing spondylitis patients treated with radiotherapy for this benign condition [14] and a number of studies using cancer registry data that have limited information on radiotherapy, typically just yes/no [15,16].

In adults there is one small study of thirteen *bone and soft tissue* sarcomas in women who had radiotherapy for breast cancer where there was some evidence of a dose–response relationship [11]. All the cases were exposed and the sarcomas occurred in on near the radiation field with the lowest estimated dose to the site of the sarcoma being 11 Gy. In the early study of cervical cancer patients (1988), which found no evidence of an increased risk of bone sarcoma, there was also no evidence of an increased risk of soft tissue sarcoma [10]. More recent registry based studies, however, do find evidence of increased risks of soft tissue sarcoma after adulthood radiotherapy, particularly after breast cancer [16-19].

We supplemented these adulthood studies with a systematic evaluation of second sarcomas after any first cancer in the US Surveillance, Epidemiology and End Results (SEER) 9 cancer registries [20]. The key advantages of the SEER registries for evaluating rare second cancers like sarcoma are the large population with long-term follow-up and systematic reporting of second cancers. In this analysis we included one year survivors diagnosed with a first cancer age 20–79 years between 1973 and 2008. Patients with a first primary bone or soft tissue sarcoma were excluded because of the difficulty of classifying second primary sarcomas. Second sarcomas were included if they were diagnosed before age 85 years. We compared the observed number of bone or soft tissue sarcomas with the expected number in the general population, which was estimated using age and sex specific incidence rates. Standardized incidence ratios (SIRs) were then calculated as the observed divided by the expected number of second sarcomas for patients who received radiotherapy and those that did not. We report results for all first cancers combined, as well as by type of first cancer and also examine patterns of SIRs according to age at and time since first cancer diagnosis.

In approximately 1.9 million 1+ year adulthood cancer survivors there were 314 second *bone* sarcoma and 1342 second *soft tissue* sarcomas diagnosed after an average follow-up of 13 years (Table 2). In general these adult cancer survivors had a small but significantly increased risk of developing a sarcoma in the bone or soft tissue compared to the general population even if they did not receive radiotherapy (SIR = 1.23 for bone cancer and 1.16 for soft tissue cancer, p < 0.05). For both types of sarcoma the SIRs were further elevated for the patients who had received radiotherapy, and this difference increased with increasing time since diagnosis of the first cancer (Table 2). By 15+ years after the first cancer diagnosis the SIRs for bone sarcoma were 4.35 in the radiotherapy group compared to 0.94 in the non-radiotherapy group, and for soft tissue sarcoma they were 2.20 and 1.21, respectively. The patients treated at a younger age also had higher SIRs than those treated at older ages (Table 3). For those diagnosed before age 40 the SIRs for bone sarcoma were 4.40 for the radiotherapy group compared to 1.31 in the non-radiotherapy group, and for soft tissue sarcoma they were 5.32 and 2.25, respectively.

**Table 2:** Standardized Incidence Ratios (SIRs) for subsequent primary sarcoma after any 1^st^ cancer in adulthood according to radiotherapy and time since first cancer diagnosis, SEER 9 1973-2008

	**Bone sarcoma**				**Soft tissue sarcoma**			
**Time since 1st**	**Radiotherapy**		**No radiotherapy**		**Radiotherapy**		**No radiotherapy**	
**Cancer diagnosis**	**Obs**	**SIR**	**Obs**	**SIR**	**Obs**	**SIR**	**Obs**	**SIR**
1-4 yrs	34	1.49*	77	1.32*	135	1.22*	311	1.15*
5-9 yrs	43	2.74*	56	1.24	185	2.22*	271	1.21*
10-14 yrs	23	3.23*	34	1.31	78	2.04*	141	1.06
15+ yrs	23	4.35*	24	0.94	62	2.20*	159	1.21*
Total	123	2.42*	191	1.23*	460	1.77*	882	1.16*

*p < 0.05. Obs-observed number of subsequent primary sarcomas. Analysis includes adulthood (age 20–79 years at diagnosis) 1+ year survivors of any 1^st^ cancer (excluding bone sarcoma or soft tissue sarcoma, respectively). Follow-up for second primary bone or soft tissue sarcoma continued to age 85 years. Patients with unknown radiotherapy were excluded.

**Table 3:** Standardized Incidence Ratios for subsequent primary sarcoma after any 1^st^ cancer in adulthood according to radiotherapy and age at 1^st^ cancer diagnosis, SEER 9 1973-2008

	**Bone sarcoma**				**Soft tissue sarcoma**			
**Age at 1st**	**Radiotherapy**		**No radiotherapy**		**Radiotherapy**		**No radiotherapy**	
**Cancer diagnosis**	**Obs**	**SIR**	**Obs**	**SIR**	**Obs**	**SIR**	**Obs**	**SIR**
20-39 yrs	13	4.40*	10	1.31	52	5.32*	58	2.25*
40-59 yrs	42	3.71*	40	1.23	129	2.41*	219	1.37*
60-79 yrs	40	2.60*	57	1.31	177	1.83*	302	1.15*

*p < 0.05. Obs-observed number of subsequent primary sarcomas. SIR-standardized incidence ratio. Analysis includes adulthood (age 20–79 years at diagnosis) 1+ year survivors of any 1^st^ cancer (excluding bone sarcoma or soft tissue sarcoma, respectively). Follow-up for second primary bone or soft tissue sarcoma continued to age 85 years. Patients with unknown radiotherapy were excluded.

In general for each of the first cancer sites that we examined the SIRs for subsequent sarcomas were higher in the patients who were treated with radiotherapy than in the patients who did not receive radiotherapy (Table 4). There were some interesting exceptions, however. For example, there was no evidence of an increased risk of bone sarcoma after radiotherapy for laryngeal or lung cancer, nor for prostate or testicular cancer. For soft tissue sarcoma there was no clear evidence of increased risks after radiotherapy for laryngeal or brain/CNS cancers. These site-specific observations warrant further investigation.

**Table 4:** Standardized Incidence Ratios for subsequent primary sarcoma according to type of first cancer and radiotherapy, SEER 9 1973-2008

	**Bone sarcoma**				**Soft tissue sarcoma**			
	**Radiotherapy**		**No radiotherapy**		**Radiotherapy**		**No radiotherapy**	
**1st cancer type**	**Obs**	**SIR**	**Obs**	**SIR**	**Obs**	**SIR**	**Obs**	**SIR**
Oral Cavity and Pharynx	9	7.61*	1	0.49	12	1.95*	8	0.75
Rectum and Anus	6	5.15*	6	1.77	12	1.82	18	0.96
Larynx	2	1.62	2	3.80	6	0.87	3	1.06
Lung and Bronchus	0	0.00	4	1.48	12	3.44*	19	1.30
Female Breast	21	2.59*	21	1.28	104	2.67*	101	1.29*
Cervix Uteri	6	6.62*	1	0.71	10	2.38*	6	1.04
Corpus Uteri	12	3.96*	3	0.62	27	1.89*	29	1.25
Ovary	3	13.99*	2	1.45	6	6.25*	9	1.44
Prostate	8	1.09	19	1.38	89	1.69*	97	1.05
Testis	0	0.00	0	0.00	9	2.60*	8	2.78*
Brain and CNS	3	10.47*	0	0.00	4	3.66*	3	3.44
Thyroid	4	4.12*	2	0.91	11	2.63*	10	1.04
Hodgkin Lymphoma	3	4.28	3	6.37*	22	8.71*	7	3.72*
Non-Hodgkin Lymphoma	10	9.00	7	2.92*	11	1.96	17	1.36
Leukemia	1	17.60	5	3.09*	1	4.17	10	1.16

*p < 0.05. Obs-observed number of subsequent primary sarcomas. SIR-standardized incidence ratio. Analysis includes adulthood (age 20–79 years at diagnosis) 1+ year survivors of the specified 1^st^ cancer type. Follow-up for second primary bone or soft tissue sarcoma continued to age 85 years. Patients with unknown radiotherapy were excluded.

## **Lower-dose radiation exposure (<5 Gy)**

The Life Span Study of the Japanese atomic bomb survivors is the most informative study of the relationship between lower dose radiation exposure (<5 Gy) and sarcoma. As well as being a large cohort with long-term follow-up it includes exposure at all ages. The latest report from this study examined the risk of all sarcomas (including those occurring at organ sites) and reported (for the first time) a significantly increased risk with increasing dose that was very similar in magnitude to the risk for other types of solid cancer, but much higher than the estimates from the studies of fractionated high-dose radiotherapy described above (Excess Relative Risk/Gray (ERR/Gy) approximately 5 for exposure at age 10) [21]. The mean dose in this cohort is only about 0.2 Gy. The ERR/Gy decreased with increasing age at exposure, which is also the pattern commonly observed for other solid cancers and may be due to higher levels of cell turnover at younger ages. In general there is no evidence of increased sarcoma risks in patients treated with lower-dose radiotherapy (<5 Gy) but the statistical power in many of these studies to detect small excess risks is generally low due to small numbers [22-24].

## **Internal radiation exposure**

There is extensive evidence that high-dose exposure to radionuclides results in radiation-induced bone sarcomas. Key studies include the follow-up of radium dial painters [25], cohorts of patients treated with 224-radium for benign conditions such as bone tuberculosis [26], and a study of nuclear workers at the Russian Mayak facility who were exposed to high-doses of plutonium, which concentrates in the bone [27]. There is also evidence that internal radiation exposure induces soft tissue sarcomas from long-term follow-up of patients treated with thorotrast [28]. As mentioned above these studies have previously been reviewed extensively [3].

## **Genetic susceptibility**

Sarcoma development is not only influenced by dose related radiation exposure, but also by genetic susceptibility. Several rare familial genetic syndromes, including familial gastrointestinal stromal tumor syndrome (GIST), Li-Fraumeni syndrome, retinoblastoma, Werner syndrome, Neurofibromatosis Type 1, Costello Syndrome, and Nijmegen breakage syndrome are associated with increased risk of bone or soft tissue sarcoma, along with multiple other tumors [29-31]. The question of whether individuals with these heritable syndromes are more susceptible to the effects of ionizing radiation than normal individuals is of interest, particularly in the context of high doses of radiotherapy. As early as the 1970’s, a marked increase in radiosensitivity was noted in clinical reports and cell-based studies of Ataxia Telangiectasia (A-T), a familial syndrome with a predisposition to develop lymphomas and leukemia [32,33]. For sarcoma-related syndromes, however, strong evidence for radiosensitivity has only been observed for Nijmegen breakage syndrome (NBS), which is primarily associated with lymphopoetic tumors [34-36]. Significantly more x-ray induced chromosomal damage has been observed in NBS lymphocytes and fibroblasts than in normal cells [36], lymphoblastoid cell lines from NBS patients show markedly poorer survival following exposure to radiation compared to those from normal individuals [37], and case-reports of severe adverse reaction to radiation therapy have been reported in a clinical setting [38]. Possible increased radiosensitivity has also been reported in cell-based experiments of Li-Fraumeni syndrome and retinoblastoma [39,40]. In human studies, although a recent cohort study of mortality from subsequent malignancy in retinoblastoma patients did not detect a significant interaction between hereditary status and treatment with radiotherapy (p = 0.12), a large proportion of the sarcomas in the irradiated patients were in the radiation field [41]. A similar finding was observed in a study of Li-Fraumeni family members in which 50% of the second sarcomas occurred in the radiotherapy field [42]. A study examining age of onset of osteosarcoma following retinoblastoma also suggested that the latency period between radiotherapy and osteosarcoma is approximately 1.3 year shorter inside than outside the radiation field [43].

Based on the evidence up to 1998, the International Commission for Radiation Protection concluded that given the high risk of spontaneous cancer in familial disorders, doses of radiation in the order of ≤0.1 Gy were unlikely to impact significantly on life-time cancer risk in an affected individual [34]. However, this relative risk could become important at high doses (5 + Gy), such as those experienced in radiotherapy. Given the paucity of evidence accumulated since then, particularly in human studies, these conclusions remain essentially unchanged.

As described in the paragraphs above, a few rare genetic variants associated with human cancer susceptibility syndromes appear to increase radiosensitivity in individuals with certain hereditary cancer syndromes. However, these syndromes affect only a small proportion of the general population. Very little empirical evidence exists to date regarding whether common genetic variants confer an increased risk of radiation-related cancer in general., Given that multiple genetic pathways including DNA damage repair, radiation fibrogenesis, oxidative stress, and endothelial cell damage have been implicated in studies of radiosensitivity [44], at least some part of the genetic contribution defining radiation susceptibility is likely to be polygenic, with elevated risk resulting from the inheritance of several low penetrance risk alleles (the “common-variant-common-disease” model). Identifying this variation in human populations is not straightforward given that studies would require large sample sizes and high-quality radiation exposure information, with sufficient power to adequately address variation in demographic and treatment factors. Regardless, studies directed at increasing understanding of susceptibility to radiotherapy-related cancers could be important in identifying high-risk individuals and may lead to clinical benefits if radiation exposures could be reduced in these patients.

## **Radiation Signatures**

Given that exposure to ionizing radiation has been long associated with increased risk of sarcoma [3], there has been considerable interest in identifying a “radiation signature” or in other words a genetic expression profile that can differentiate between radiation-related tumours and sporadic tumours. Studies searching for a radiation signature have generally used some modification of the 1948 Cahan criteria for classifying sarcomas as radiation-related: the tumor must be in the irradiated field, histologically different from the primary cancer, and have a latency period of at least five years [45]. While satisfying these criteria is likely to result in a high probability that the sarcoma is radiation-related, it does not guarantee that it was radiation-induced. Nonetheless, we maintain the terminology “radiation-induced” and the comparison group of “sporadic sarcomas” in this article for succinctness.

Early work in search of a radiation signature for sarcoma used conventional cytogenetic analysis to identify large-scale chromosomal abnormalities. Two small studies without a comparison group used trypsin-giemsa banding [46] and comparative genomic hybridization [47] to detect a variety of large scale abnormalities – however, only the loss of material on chromosomes X and 13 was observed using both techniques. More recent studies using polymerase chain reaction followed by direct sequencing were able to examine mutations in specific genes: a high rate of *TP53* mutation was observed in radiation-induced sarcomas compared to sporadic sarcomas (88% vs 20%, and 58% vs. 16.8% for two different series) [48,49]. While both studies focused on sarcoma subsequent to a variety of primary tumours, 12/36 of cases in the Gonin-Laurent study were secondary to retinoblastoma, a syndrome caused by mutations in the *RB1* gene. The authors later examined the status of *RB1*, *TP53* and four additional genes involved in their regulation, and hypothesized that inactivation of the p53 allele in 12/36 cases was due to irradiation rather than *RB1* loss. They also noted that neither gene pathway was inactivated in 40% of radiation-induced tumours, suggesting the presence of further unidentified pathways involved in radiation carcinogenesis [50]. A recent study by Hadj-Hamou et al. was able to address multiple biological pathways using an agnostic and generalizable microarray analysis approach [51]. The authors identified a signature of 135 genes from a learning/training set of 12 radiation-induced and 12 sporadic sarcomas, and were able to discriminate radiation-induced from sporadic sarcomas in an independent set of 36 sarcomas of various histologies with 96% sensitivity and 62% specificity. Examination of the gene pathways suggested a particular role for mitochondrial genes and genes involved in detoxification or antioxidant functions, suggesting that mitochondrial dysfunction and chronic oxidative stress could be hallmarks of radiation-induced tumours. While the results of this study require replication, this approach may be useful in identifying distinct molecular signatures for radiation-induced sarcoma, which in turn could provide clues regarding the molecular etiology of these tumors. It will be particularly important to test the approach in different settings of radiation dose and dose fractionation, while taking into account the potential roles of chemotherapy and tumor heterogeneity.

## **Summary**

In summary, radiation exposure remains one of the few established risk factors for both bone and soft tissue sarcomas. Similar to many other cancers children have the highest risks of developing a radiation-related sarcoma. Recent new findings from the Japanese atomic bomb survivors suggest, for the first time, that the risk is not limited to high-dose exposures (10 + Gy), but at lower-dose levels a radiation-related sarcoma would be an extremely rare event. While there is evidence that individuals with certain rare familial genetic syndromes predisposing to sarcoma, particularly Nijmegen Breakage Syndrome, and possibly Li-Fraumeni syndrome and retinoblastoma, are particularly sensitive to the effects of high dose radiation, it is unclear whether this is also true in low-dose settings. Efforts to limit unnecessary high-dose radiation exposure, particularly in children, remain important given the high fatality rates associated with this disease.

## Competing interest

The authors declare that they have no competing interests.

## Authors’ contributions

AB, AK and PR conducted the literature reviews and contributed to drafting of the manuscript and reviewed the manuscript. AB conducted the statistical analyses. All authors read and approved the final manuscript.
